# O-arm navigation-guided uni-portal non-coaxial spinal endoscopic surgery for the precise treatment of far-out syndrome—a case report and literature review

**DOI:** 10.3389/fsurg.2026.1763225

**Published:** 2026-06-17

**Authors:** Kai Lv, Dong Zhang, Xinhe Wang, Qiugen Wang, Zhichao Ge, En Song, Hao Sun, Mencheng Yin, Jiajun Wu, Shuqiang Wang, Jianmin Li

**Affiliations:** 1 Department of Spine Surgery, Yueyang Hospital of Integrated Traditional Chinese and Western Medicine, Shanghai University of Traditional Chinese Medicine, Shanghai, China; 2 Department of Orthopedics Center, Yueyang Hospital of Integrated Traditional Chinese and Western Medicine, Shanghai University of Traditional Chinese Medicine, Shanghai, China; 3 Department of Dermatology, Yueyang Hospital of Integrated Traditional Chinese and Western Medicine, Shanghai University of Traditional Chinese Medicine, Shanghai, China; 4 Department of Sports Medicine, The First Affiliated Hospital of Kunming Medical University, Kunming, China

**Keywords:** case report, extraforaminal stenosis, far-out syndrome, minimally invasive spinal surgery, O-arm navigation, uni-portal non-coaxial spinal endoscopic surgery

## Abstract

Far-out Syndrome is a rare condition involving extracanal compression of the L5 nerve root at the far-lateral region where it passes between the L5 transverse process and the sacral ala. The limited availability of advanced imaging modalities constrains the detection rate of far-out Syndrome. Correspondingly, a standardized surgical approach has not yet been fully established within the medical community. In this case we firstly introduce the combined use of O-arm navigation and uni-portal non-coaxial spinal endoscopic surgery to manage far-out Syndrome. A 67-year-old male presented to us with chief has complaint of left lower limb radiculopathy and numbness and paresthesia for 1 year. The patient underwent nerve root block and endoscopic decompression right after the onset of these symptoms with little improvement. His preoperative computed tomography scan revealed compression of left L5 nerve root in the extraforaminal region and confirm the diagnosis of far-out Syndrome. Uni-portal non-coaxial spinal endoscopic surgery decompression via paraspinal approach was performed for his under O-arm navigation. The immediate relief of symptoms post-surgery and the absence of significant recurrence during the 6-month follow-up period suggest that this surgical approach is a safe and reliable treatment option for far-out Syndrome.

## 1 Introduction

Far out syndrome, also known as distal syndrome, is a relatively underdiagnosed spinal disorder (1). Despite over four decades since its initial conceptualization (2), there is a scarcity of clinical literature currently available on this condition. The most fundamental pathological change in FOS is the L5 nerve root compressed by multiple conditions in the extraforaminal region between the L5/S1 intervertebral foramen and the lumbosacral tunnel formed by the L5 transverse process (TP) and the sacral ala (SA) (3). Due to the presence of compression of the L5 nerve root, FOS is frequently misdiagnosed in clinical practice due to its symptomatic similarity to common conditions such as Lumbar Disc Herniation or Lumbar Spinal Stenosis. The osseous nature of the compression in FOS also results in a poor response to conservative treatment among affected individuals. The confluence of low incidence and detection rates contributes to the absence of a consensus on surgical management for FOS. Consensus on surgical treatment for FOS has eluded orthopedic surgeons, hindered by the condition's low incidence and detection rates. Presently available surgical interventions encompass traditional open procedures as well as minimally invasive techniques like Unilateral Biportal Endoscopy (UBE) that have gained traction in recent years (1, 3, 4). Nevertheless, FOS remains a clinical challenge.

Uni-portal non-coaxial spinal endoscopic surgery (UNS) is an innovative surgical approach (5). This surgical technique creates a working channel through a single incision, and both the surgical instruments and the endoscope are inserted into the channel from opposite ends of the incision. The advantage of this technique is that by exchanging the positions of the viewing endoscope and the surgical instruments, the surgical field of view can be made completely consistent with the reachable range of the instruments (6–9).

In this case report, we present a 67-year-old male patient who presented with a 1-year history of low back pain, hip pain, and radiating pain in the left lower limb. Over this period, he experienced recurrent exacerbations that severely limited his mobility, culminating in multiple nerve root blocks and two surgeries (Figure 1). The diagnosis of FOS was confirmed through comprehensive imaging and an L5 nerve root block. Subsequently, successful decompression of the L5 nerve root was achieved using UNS under O-arm navigation.

**Figure 1:**
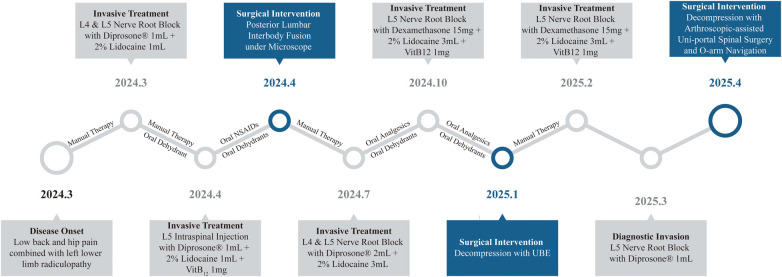
Timeline of disease onset and major treatments.

## 2 Case report

A 67-year-old male described in this article was transferred to our hospital and admitted to our department on March 18, 2025. His chief complaint was low back and hip pain combined with left lower limb radiculopathy that began over a year ago [numerical rating scale [NRS], 9/10, Oswestry Disability Index [ODI], 94%]. The body mass index was 22.86 kg/m^2^ (70 kg/175 cm). Medical history indicates well-controlled Grade 1 hypertension. The patient exhibits restricted functional capacity, unable to sustain ambulation or upright positioning beyond 3 min. On physical examination, straight left leg raise test elicited sharp L5 radiating pain at 30° of elevation. He had previously undergone two surgical interventions before this hospitalization. Posterior Lumbar Interbody Fusion (PLIF) was performed in April 2024, leading to initial pain reduction (NRS 2/10) lasted ≤ 2 months, with subsequent rebound (NRS 7/10). The anteroposterior and lateral lumbar radiographs obtained after PLIF demonstrate well-positioned implants with no evidence of significant disc space narrowing or compression (Figure 1A). The progressively worsening symptoms after recurrence drove the patient to seek another opinion at other medical institutions for further management. He underwent multiple rounds of nerve root blocks and intraspinal injections during the subsequent months. Few improvements were achieved. Decompression with UBE techniques in January 2025 improved the radiculopathy and motor weakness. While severe iatrogenic mechanical low back pain and similar radiculopathy emerged again around 7 days after the discharge (NRS 9/10). His severe pain did not improve with physiotherapy and other conservative management like acupuncture and orthopedic manual therapy.

Upon the patient's initial visit to our hospital, they brought magnetic resonance imaging (MRI) scans obtained from an external facility. Our initial assessment prioritized evaluating for significant intraspinal compression, however, radiological review of these images revealed the degree of observed compression was inconsistent with the severity of the patient's symptoms. Based on the cumulative medical history and imaging findings detailed previously, we have ruled out diagnoses of spinal stenosis, lumbar disc herniation, and vertebral compression fractures. Through multidisciplinary consultation with diagnostic radiologists and evidence-based literature review (10–12), another computed tomography (CT) scans were taken (Figure 2). Reconstruction of CT images confirmed the presence of a Castellvi Type 2a lumbosacral transitional vertebra (LSTV) (13) (Figure 2C). Given the complicated surgical history, we performed another selective nerve root block for the left L5 nerve root for diagnostic purpose (14). Short-term pain relief after the operation confirmed that the left L5 nerve root is the source of the pain. To this extent, we have confirmed the diagnosis of FOS in this case.

**Figure 2:**
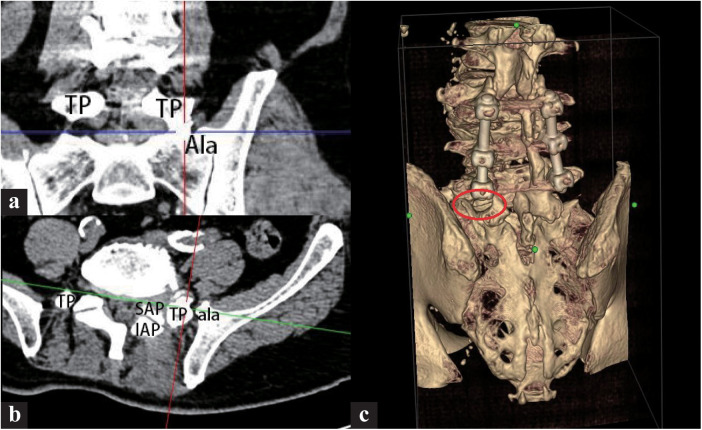
Computed tomography scans confirmed the presence of extraforaminal entrapment and the diagnosis of Far-out syndrome. **(a)** Coronal lumbar spine CT before surgery. **(b)** Axial lumbar spine CT before surgery. **(c)** Lumbar spine CT reconstruction before surgery.

After thorough reevaluation of sites of previous UBE decompression and considering the patient's persistent dedication to achieving symptom resolution, we opted for complementary UNS via paraspinal approach under O-arm navigation to decompress his left L5 nerve root at the extraforaminal zone.

## 3 Surgical approach and procedure

Following the induction of general anesthesia and confirmation of adequate anesthetic depth, the patient was positioned prone on a carbon fiber surgical table with the abdomen suspended to reduce intra-abdominal pressure and minimize intraoperative bleeding. After thorough disinfection, the dynamic reference array frame was fixed onto the specific lumbar bridge that we used, which was 3D printed. Following this, intraoperative CT scan was obtained using the O-arm navigation system.

The surgeon performed a preliminary localization of the operative segment via surface anatomical landmarks, followed by precise identification of the affected intervertebral space using a Kirschner wire under O-arm fluoroscopic guidance. The navigated probe was placed on the skin and reformatted images on the workstation give an idea about its inclination and projected trajectory. With the help of navigation, one can accurately point towards the extraforaminal region while avoiding the iliac crest. The needle was inserted along the projected trajectory of the navigation into the intervertebral space near the medial side of the pedicle and advanced to the proximal articular process lateral to the lamina. Upon contacting the bony structure, the needle was immediately removed.

A 1.5 cm incision was made along the lateral side of the articular process and gradually dilated the surgical channel. After dilation, the dilators were removed, and these incisions penetrated the fascia to maintain continuous outflow of the irrigation saline during the surgery. At this point, the role of navigation is crucial, as the instruments used for decompression must reach the target point without any obstruction.

In this case, the chief surgeon was right-handed, so the endoscope was inserted from the cranial side of the incision, while the navigated probe was inserted from the caudal side of the incision. The 30° spinal endoscope was introduced under real-time guidance of the O-arm. The navigated probe was placed directly on the bony landmarks to identify the TP, superior articular process (SAP), and superior sacral notch (Supplementary Figure S1). Then the light source and irrigation system were connected, revealing the articular capsule of the articular process.

The radiofrequency probe was inserted from the caudal side of the incision. Navigation assisted in identifying the target site, first, clear the soft tissue and alternately use the electrosurgical cutting and coagulation functions of the radiofrequency probe to prevent soft tissue bleeding, and then outlining the bony surface of the SA, SAP lateral aspect of sacral vertebra, and the lower border TP of the lumbar 5 vertebra (Figure 3). Due to the abundant soft tissue and complex anatomical structures in the area, a navigation probe was used to periodically reconfirm the anatomical structures to ensure that operations were performed on the bony surface and to prevent injury to nerves and blood vessels.

**Figure 3:**
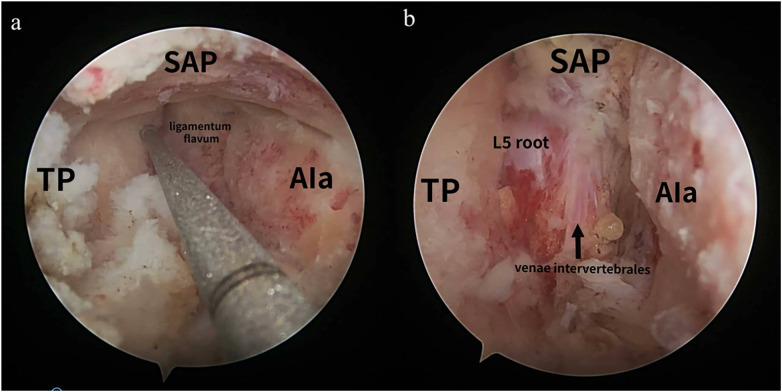
Intraoperative findings. **(a)** Key bony landmarks were ultimately identified following layer-by-layer dissection of the soft tissues under endoscopic approach. **(b)** Endoscopic view of the decompressed area.

We chose a high-speed drill and an ultrasonic bone scalpel, and it is recommended to start from the target site using the “circular method” to remove the lower border TP of the lumbar vertebra 5, the upper portion of the SA, and SAP lateral aspect of sacral 1 vertebra. The high-speed drill can reduce bleeding from the cancellous bone, while the ultrasonic bone scalpel offers higher safety. Once the bony resection is completed, the cranial portion of the ligament is detached and reflected from the facet joint and TP using a nerve hook, followed by resection with a Kerrison rongeur. After resecting the ligament, the exiting nerve root is easily identified. At this stage, potential sources of bleeding may arise from the complex plexus of veins and adhesions around the neurovascular structures, and thorough hemostasis of the soft tissue is achieved using radiofrequency ablation. The exposed nerve root appeared relaxed and tension-free, indicating adequate decompression. Upon completion of the procedure, under endoscopic visualization, nerve root relaxation was reconfirmed. The endoscope was then withdrawn, a negative pressure drainage ball was placed, the incision was sutured, and a sterile dressing was applied.

On the first day after surgery, the patient was able to ambulate normally and participated in tailored massage and rehabilitation therapy at our hospital. The patient's postoperative NRS score decreased from 9/10 preoperatively to 3/10. The pain experienced by the patient was mainly from the lumbar incision and the muscles of the surgical approach, while lower limb pain and radiating pain were completely relieved. One-month follow-up imaging indicated improvement in the LSTV (Figure 4). A six-month follow-up confirmed no recurrence of symptoms, and the ODI score decreased from 94% preoperatively to 12%.

**Figure 4:**
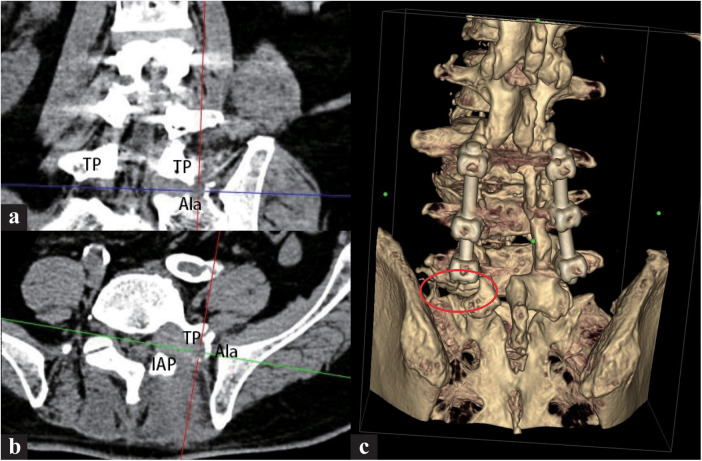
One-month postoperative follow-up CT images: **(a)** coronal view of the surgical site; **(b)** axial view of the surgical site; **(c)** CT reconstruction of the lumbar spine at the surgical site.

## 4 Discussion

FOS, first described in 1984 by the American spinal specialist Wiltse, is a pathological condition characterized by compression of the L5 nerve root within the extraforaminal zone (2). Multiple factors like osteophyte hyperplasia or ligamentous hypertrophy, may contribute to this compression. When nerve compression results from a congenital anomalous connection between L5 TP and SA forming a pseudoarticulation, this condition may also be described as Bertolotti syndrome (4).

Due to an incomplete understanding of pathology and few cohort observations from bedside, the current disease burden of FOS remains unquantifiable. The estimated prevalence of LSTV may reach as high as 20% in the general population, though the proportion that develops clinically significant symptoms remains poorly defined (15). In contrast to East Asia, where awareness of FOS is well-established, screening in other regions remains under-prioritized. This is corroborated by the annual detection of over 50 cases at single institutions in countries such as South Korea (16) and Japan (17), indirectly affirming a high local prevalence. In the absence of a correct identification of this condition, the high frequency of failed symptom relief or rapid recurrence following aimless surgical interventions constitutes a major persistent clinical challenge in managing FOS. However, our clinical observation confirms its profound impact on patients. Intractable chronic pain kept haunting the patient described in this article, which drive him subjected to over a dozen surgical interventions.

Definitive diagnosis of FOS serves as the foundational gatekeeper to targeted therapeutic strategies. Clinically, FOS exhibits high resemblance to other kinds of refractory low back pain or sciatica, necessitating careful differential diagnosis among these entities (18). In the diagnostic limbo preceding FOS confirmation, other patients typically undergo burdensome cycles of redundant imaging, futile nonoperative management and ultimately unnecessary operations directed at erroneous pain generators, most commonly mistaken L4-L5 disc pathologies. Therefore, evaluation of extraforaminal compression and conventional intraspinal pathologies with imaging techniques can enhance detection rate of FOS. The imaging evaluation for FOS faces limitations in regions with underdeveloped healthcare infrastructure. Though transverse CT scan lacks diagnostic values, CT of 3D-reconstruction remains the universally accessible gold standard for diagnosis. A TP-SA interval ≤ 3 mm, osteophyte formation at the compression site, subchondral cysts, and articular surface sclerosis collectively guide definitive FOS identification (11). Besides, MRI enables direct visualization of injury severity in compressed neural tissues. Oblique coronal T2-weighted sequences reveal a foraminal spinal nerve angle exceeding the contralateral asymptomatic side by >10°, while perineural deformation and edema provide definitive evidence confirming FOS diagnosis (19). Given that FOS presents with a characteristic nerve injury, diagnostic nerve root blocks further assist in precisely localizing the compressed spinal nerve segment, providing corroborative evidence to solidify the diagnosis of FOS (14). Besides, electrophysiological methods find applications in the clinical assessment in select cases (17). However, this technique remains relatively immature. The definitive diagnosis still rests squarely on radiographic imaging and confirmatory selective nerve root blocks. This situation inevitably leads to a large number of patients being missed or misdiagnosed.

Currently, no internationally standardized criteria exist for assessing the severity of FOS. Castellvi classification was established to assess LSTV, but failed to quantify neuro-compressive sequelae. Advancing validated FOS-specific metrics will enable evidence-based standardization in selecting conservative management vs. surgical intervention.

The characteristics of FOS necessitate surgical intervention for effective relief, and the development of the surgical treatment has evolved alongside a growing boosting of surgical technology. We have summarized the relevant literature regarding the surgical management of FOS in Table 1. Initially, the surgery was primarily performed with the assistance of a microscope, with both anterior (20) and posterior (21) approaches being reported. Subsequently, with the maturation of endoscopic techniques, some teams began attempting endoscopic decompression around 2009 (22). By 2016, clinical reports related to FOS generally adopted microendoscopic assistance, with a standardized posterior approach (predominantly paraspinal approach) (23, 24). In 2018, the relatively mature and stable technique of UBE was introduced into clinical practice, achieving satisfactory outcomes in the majority of cases (25). Our team has extensively studied and mastered the UBE technique, achieving excellent results in the treatment of several other diseases. A review of the literature clearly shows that the UBE technique is also applicable to FOS, a condition predominantly involving far-lateral lesions. After adequate preoperative evaluation and selection of the correct surgical approach, UBE can definitely achieve good outcomes. In this patient's case, the previous UBE procedure achieved only temporary pain relief, which should be attributed more to the lack of clarity in the preoperative diagnosis, resulting in the previous surgery focusing mainly on decompression of intervertebral or intralaminar lesions.

**Table 1:** Summary of previous literatures about Far-out syndrome.

Study	Case Number	Surgical Technique
Watanabe et al. 2025 (26)	6	Decompression via posterior approach under MES
Hu et al. 2025 (27)	5	Decompression via paraspinal approach under MES and C-arm Navigation
Choi et al. 2025 (28)	1	Decompression via paraspinal approach under MES and C-arm Navigation
Maki et al. 2025 (29)	2	Oblique LIF at L5/S1
Yang et al. 2025 (30)	16	Decompression via paraspinal approach under MES and C-arm Navigation
Kavishwar et al. 2024 (31)	1	Decompression via paraspinal approach under UBE and O-arm Navigation
Ha et al. 2024 (32)	1	Decompression via paraspinal approach under UBE and C-arm Navigation
Park et al. 2021 (1)	72 (35 reported)	Decompression via paraspinal approach under UBE and C-arm Navigation
Kanematsu et al. 2020 (33)	68 (22 reported)	Decompression and (or) transforaminal LIF via paraspinal approach under MS or MES
Hoe et al. 2019 (3)	16 (14 reported)	Decompression via paraspinal approach under UBE and C-arm Navigation
Kim et al. 2018 (25)	1	Decompression via paraspinal approach under UBE
Ikuta et al. 2018 (34)	25	Decompression via paraspinal approach under MES
Ise et al. 2016 (35)	1	Decompression and transforaminal LIF via paraspinal approach under MES
Stavrinou et al. 2016 (23)	4	Decompression via transmuscular approach under MES and C-arm Navigation
Takeuchi et al. 2015 (19)	113	/
Tschugg et al. 2015 (24)	1	Decompression via paraspinal approach under MS
Iwasaki et al. 2014 (17)	50	/
Bezuidenhout et al. 2014 (12)	14	/
Lee et al. 2014 (16)	52	Decompression via far-lateral approach under MS
Kim et al. 2013 (36)	28 (4 operated)	Decompression
Kikuchi et al. 2013 (37)	2	Decompression via extraperitoneal approach under MS for 1 patient Decompression via retroperitoneal approach under MS for 1 patient
Byun et al. 2012 (38)	18	/
Kitamura et al. 2012 (39)	1	Decompression via posterior approach and posterior LIF under MS
Sasaki et al. 2012 (40)	8	Decompression via posterior approach under MS
Nakao et al. 2010 (11)	10	Decompression under MES
Moon et al. 2010 (10)	3	/
Matsumoto et al. 2010 (41)	28	Decompression via posterior approach under MS for 9 patients Decompression via posterior approach under MES for 19 patients
Kim et al. 2009 (42)	6	/
Zhou et al. 2009 (22)	5	Decompression via intertransverse approach under MES
Ichihara et al. 2004 (43)	2	Decompression via lateral approach without MS or MS
Liu et al. 2003 (44)	1	/
Park et al. 2003 (45)	21 (16 operated)	Decompression via midline approach under MS
Matsumoto et al. 2002 (21)	4	Decompression via posterior approach under MS
Abe et al. 1997 (20)	1	Decompression via extraperitoneal approach under MS

MS, Microscope; MES, Microendoscope; UBE, Unilateral Biportal Endoscopic; LIF, Lumbar Interbody Fusion.

The O-arm can provide real-time navigation. In this case, because the lesion was located outside the spinal canal, the surgical field was prone to being obscured by structures such as the L5 transverse process, the sacroiliac ligament, and the iliac wing. Use of a C-arm would require frequent intraoperative fluoroscopy, whereas O-arm navigation allows for precise localization, real-time navigation, safe decompression, and avoidance of nerve injury in complex anatomical regions.

In 2021, the concept of UNS was first proposed, marking an innovative application of arthroscopy in spinal surgery. UNS is a continuation and upgrade of the intervertebral foraminoscopy and an improvement of the unilateral dual-channel technique. UNS combines the single-sided dual-channel double holes into a single hole, adding an endoscope to the conventional open surgery, with the advantages of open observation field, free operation space, and compatibility with a variety of spinal surgery techniques and instruments. Furthermore, UNS presents a relatively gentle learning curve, making it more accessible for surgeons with open surgery experience and reducing the likelihood of disorientation for novices (6).

The primary goal of this article is to showcase the combined use of O-arm navigation and UNS in the management of FOS. This innovative approach integrates advanced imaging technology with minimally invasive surgical techniques to enhance precision and outcomes in treating FOS, a condition characterized by nerve compression in the far lateral zone. Through this demonstration, the article aims to highlight the benefits of combining these 2 technologies, offering improved visualization, realtime guidance, enhanced precision, and a safer, more effective intervention for patients with FOS.

The UNS procedure also has its own limitations. Although it requires only one incision, this incision is usually slightly larger than that of the UBE procedure, resulting in greater exposure of soft tissue. Postoperative wound healing around the surgical incision may be complicated by issues such as poor healing or fat liquefaction. On the other hand, due to the relatively short period of application of the UNS procedure, relevant operational standards have not yet been established. This has, to some extent, limited the widespread adoption of the UNS procedure.

In conclusion, combining O-arm navigation with UNS for the management of FOS makes this decompression surgery highly precise and meticulous. This helps to avoid the complications associated with the paraspinal UNS approach and also reduces the learning curve of UNS.

## Data Availability

The original contributions presented in the study are included in the article/Supplementary Material, further inquiries can be directed to the corresponding authors.
