# Supplementary figures and images for: O-arm navigation-guided uni-portal non-coaxial spinal endoscopic surgery for the precise treatment of far-out syndrome—a case report and literature review
Source: Front Surg. 2026 Jun 17;13:1763225. doi: 10.3389/fsurg.2026.1763225 (PMC13319011; doi:10.3389/fsurg.2026.1763225)

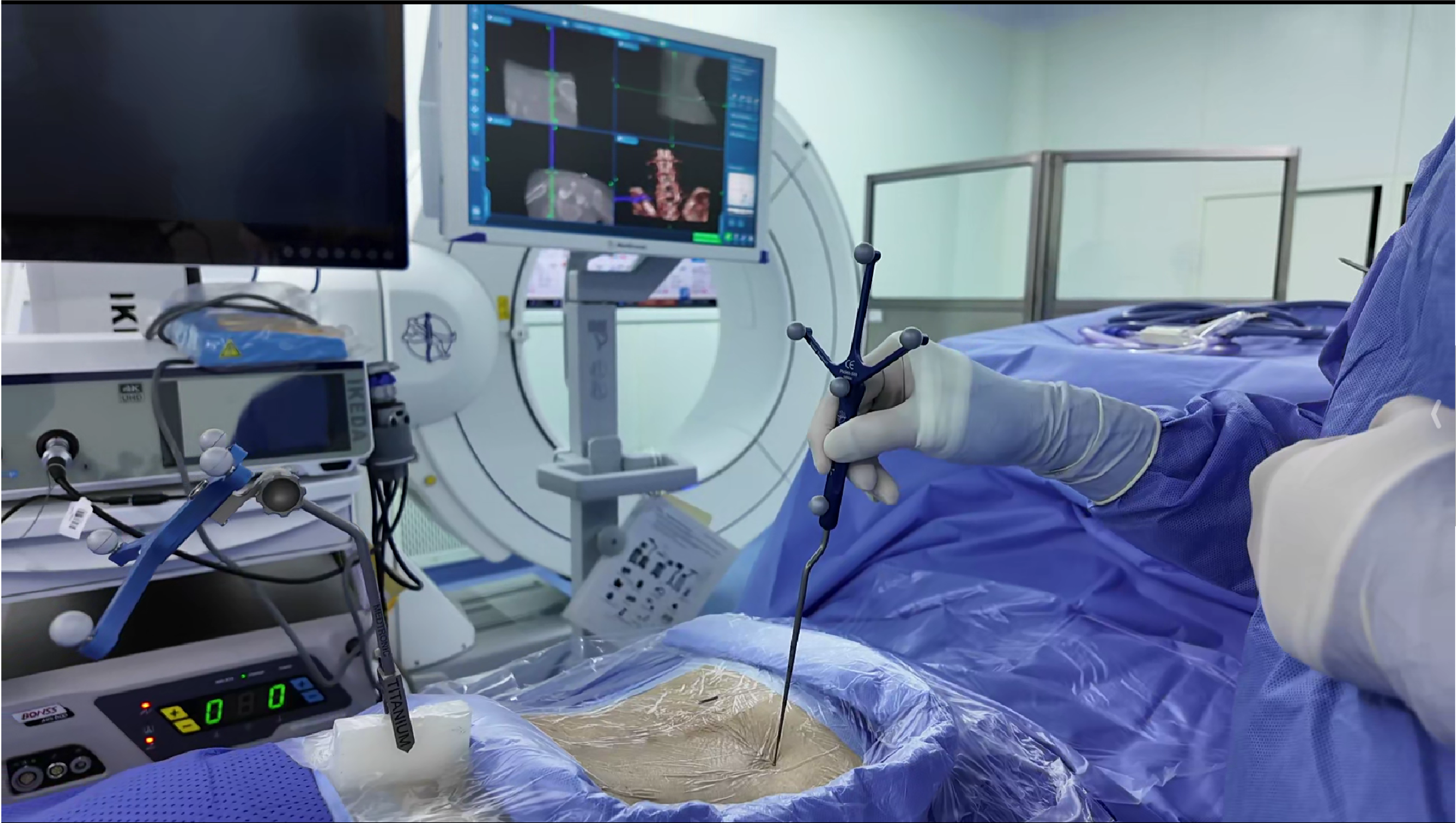

Supplement: Supplementary file 1 [file Image1.tif]
